# Evaluation of Predation on Phytophagous Insects by a Phytozoophagous Mirid Bug, *Apolygus lucorum*

**DOI:** 10.3390/insects17040397

**Published:** 2026-04-07

**Authors:** Lili Wang, Baoyou Liu, Kongming Wu

**Affiliations:** 1 Yantai Academy of Agricultural Sciences, Yantai 265500, China; wsp0127@163.com (L.W.); baoyou1022@163.com (B.L.); 2 State Key Laboratory for Biology of Plant Diseases and Insect Pests, Institute of Plant Protection, Chinese Academy of Agricultural Sciences, Beijing 100193, China; 3 National Center of Technology Innovation for Comprehensive Utilization of Saline-Alkali Land, Dongying 257300, China

**Keywords:** mirid bug, functional response, COI molecular detection, predatory behavior

## Abstract

*Apolygus lucorum*, a phytozoophagous mirid bug, plays an important role in the species interactions within fruit tree and cotton ecosystems. Previous research has mainly focused on the phytophagous damage that it causes to crops, while its role as a predator of arthropods remains poorly understood. In this study, we systematically investigated the functional responses of *A. lucorum* to three crop pests: eggs of *Helicoverpa armigera*, nymphs of *Aphis gossypii*, and nymphs of *Bemisia tabaci*. The results show that the predatory behavior of *A. lucorum* towards all three prey species followed a Holling type II functional response model. Predatory performance varied significantly depending on prey species, developmental stage, and sex of the mirid. The theoretical maximum predation rate was highest for *A. gossypii* (833.33 individuals/day) and lowest for *B. tabaci* nymphs. Adult mirids and older nymphs (4th instar) exhibited higher predation rates than younger nymphs. Field-collected *A. lucorum* from Bt cotton fields were analyzed using molecular diagnostics, and the result confirmed natural predation on *A. gossypii*, which was consistent with observed pest occurrence patterns in the field. Overall, this study clarifies the prey selectivity and stage-dependent predatory strategies of *A. lucorum*, providing insights into its trophic flexibility as a facultative predator. These findings contribute to a more comprehensive understanding of its ecological role in agricultural ecosystems, but do not support its use as a biological control agent given its predominantly phytophagous nature and documented pest status.

## Simple Summary

*Apolygus lucorum* is a significant agricultural pest that also exhibits predatory behavior on small arthropods. This study systematically evaluated its predatory capacity against eggs of *Helicoverpa armigera*, nymphs of *Aphis gossypii*, and nymphs of *Bemisia tabaci*. Laboratory experiments demonstrated that *A. lucorum* showed the strongest predatory preference for *A. gossypii*. Its predatory ability increased with developmental stage, and males generally exhibited higher predation efficiency than females. DNA-based gut content analysis of *A. lucorum* in Bt cotton fields confirmed its predation on *A. gossypii* in natural environments. These findings systematically elucidate the predatory characteristics and ecological adaptability of *A. lucorum*, contributing to a deeper understanding of its functional role in agricultural ecosystems.

## 1. Introduction

*Apolygus lucorum* (Hemiptera: Miridae) is a phytozoophagous pest widely distributed in agricultural ecosystems, with over 200 recorded host plants. It causes significant damage to cotton and several fruit crops including jujube, grape, and peach [1,2,3,4]. Traditionally, *A. lucorum* is unequivocally defined as a phytophagous pest. Both adults and nymphs of *A. lucorum* feed by piercing tender plant tissues such as buds, young leaves, flower buds, and immature fruits. This feeding behavior initially results in small dark-brown necrotic spots that progressively expand and coalesce as plant development continues, ultimately leading to leaf perforation, fruit deformity, and bud abscission. These symptoms severely compromise crop yield and quality, frequently resulting in substantial economic losses [5,6,7,8]. Consequently, research has predominantly focused on the population dynamics, damage mechanisms, and control strategies of *A. lucorum* [9,10,11].

However, emerging evidence from field observations and laboratory studies indicates that *A. lucorum* exhibits facultative predatory behavior. In addition to feeding on plants, it actively preys on small arthropods such as aphids, whiteflies, and lepidopteran eggs [12,13]. This mixed feeding strategy—primarily phytophagous with opportunistic zoophagy—is not uncommon among mirid bugs. For example, the tarnished plant bug, *Lygus lineolaris* (Hemiptera: Miridae), a major pest of cotton, alfalfa, and strawberry, has also been reported to prey on spider mites and aphids [14,15]. Similarly, *Adelphocoris suturalis* (Hemiptera: Miridae), in addition to its phytophagous damage to cotton, can consume eggs and early-instar larvae of *Spodoptera exigua* (Lepidoptera: Noctuidae) and *Agrotis ipsilon* (Lepidoptera: Noctuidae) [16]. Feng et al. [17] further documented that adults of *Adelphocoris lineolatus* (Hemiptera: Miridae) can prey on early-instar larvae of *Leptinotarsa decemlineata* (Coleoptera: Chrysomelidae). Collectively, these observations suggest that facultative predation represents a widespread ecological adaptation within the Miridae family, likely enhancing population stability and ecological flexibility in heterogeneous agricultural environments.

From an ecological perspective, insects possessing facultative predatory traits often function as key species in agroecosystems, contributing to the maintenance of community stability and the regulation of trophic interactions. In primarily phytophagous mirids, the expression of zoophagy is generally considered a nutritionally driven strategy, often triggered by the need for specific nitrogenous compounds or by suboptimal plant resources [18]. For *A. lucorum*, predation may occur when prey availability aligns with periods of high nutritional demand, such as during nymphal development or adult reproduction, or when preferred host plant tissues become scarce [19,20]. These insects not only directly affect prey populations through predation but may also indirectly influence plant performance and community assembly [21,22,23]. For instance, in protected cultivation systems, mirid species such as *Nesidiocoris tenuis* (Hemiptera: Miridae) and *Macrolophus pygmaeus* (Hemiptera: Miridae) have proven effective in suppressing populations of whiteflies, thrips, and spider mites, thereby modulating their seasonal abundance patterns [24,25,26]. Such findings provide important insights into the ecological functions of facultatively predatory mirids.

*A. lucorum* is one of the most widely distributed and economically damaging omnivorous mirid pests in Chinese agroecosystems. Its population has increased significantly following the large-scale adoption of Bt cotton, and it has become a major pest of cotton, jujube, grape, and other crops [2,3]. In China, *A. lucorum* populations are characterized by high densities and prolonged seasonal activity, a trend exacerbated by the expansion of horticultural crops such as fruit trees and reduced insecticide applications in Bt cotton fields, collectively driving a substantial rise in its abundance and amplifying its ecological and economic influence within agricultural ecosystems [27,28]. Understanding the conditions that trigger predation in *A. lucorum* is therefore critical for predicting its behavior in field settings.

Given the documented pest status of *A. lucorum* and the increasing recognition of its facultative predatory behavior, this study aims to characterize its predation capacity under controlled conditions while critically evaluating the extent to which such capacity translates to natural field settings. By integrating laboratory functional response assays with field molecular detection, we seek to determine whether predation by *A. lucorum* represents a functionally significant regulatory force or merely an opportunistic nutritional supplement. This distinction is essential for accurately interpreting its ecological role and avoiding overestimation of its potential as a natural enemy in integrated pest management (IPM) programs.

In this study, we investigated the predatory potential of *A. lucorum* toward three economically important pests in China: eggs of *Helicoverpa armigera* (Lepidoptera: Noctuidae), nymphs of *Aphis gossypii* (Hemiptera: Aphididae), and nymphs of *Bemisia tabaci* (Hemiptera: Aleyrodidae). Under controlled laboratory conditions, we systematically quantified the functional response of *A. lucorum* to these prey types, with particular emphasis on the effects of predator developmental stage (nymphal instars and adults), sex, prey species, and prey density on key foraging parameters including instantaneous attack rate, handling time, and estimated maximum daily consumption. In parallel, field surveys were conducted in cotton fields to monitor the seasonal dynamics of *A. lucorum* and its potential prey. Molecular gut content analysis was employed to detect predation events in field-collected *A. lucorum* individuals, providing direct evidence of its feeding on pests such as *A. gossypii* under natural conditions. By integrating laboratory experiments with field validation, this study aims to establish a comprehensive understanding of the trophic ecology of *A. lucorum*, thereby offering a scientific basis for evaluating its functional role in agricultural ecosystems and informing the development of ecologically based integrated pest management strategies.

## 2. Materials and Methods

### 2.1. Insect Rearing

The *A. lucorum* colony was established from adults collected in Langfang, Hebei Province, and maintained continuously in the laboratory on green beans sterilized with 5% sodium hypochlorite [29]. Nymphs were reared in transparent plastic containers (19.5 cm × 13.4 cm × 7.2 cm) with fresh or split green bean pods, at a 200–300 density of individuals per container. Several folded filter paper strips (1 cm × 20 cm) were placed at the bottom of each container to facilitate insect movement. The container opening was covered with medical gauze and secured with the lid to prevent escape, and a square opening (7 cm × 7 cm) was cut in the lid for ventilation. Upon reaching adulthood, adults were also fed with green beans, and the ends of the beans were cut at a slant to serve as an oviposition substrate. A cotton ball soaked with 10% honey–water solution was placed on the gauze cover to provide supplement nutrition. After oviposition, green beans bearing egg masses were placed in a ventilated area for 4–5 days and then transferred to clean containers. Egg hatching was monitored daily. Newly hatched nymphs from the same day were transferred to another clean container with fresh green bean pods and reared as described above for subsequent generations. Rearing conditions were maintained at 26 ± 1 °C, 70 ± 5% (RH), and a photoperiod of 14 L/10 D.

The *H. armigera* colony was established from individuals collected in cotton fields in Xinxiang, Henan Province, and maintained in the laboratory on an artificial diet. Adults were provided with 10% sugar solution as a nutritional supplement. Rearing methods followed Liang et al. [30]. Rearing conditions were 26 ± 1 °C, 70 ± 5% (RH), and a photoperiod of 16 L/8 D. 

The experimental populations of *A. gossypii* and *B. tabaci* were collected from cotton fields in Langfang and were used directly in experiments without laboratory rearing.

### 2.2. Laboratory Predation Assays

To comprehensively evaluate the predatory capacity of *A. lucorum* across different developmental stages, we selected the 2nd instar nymphs, 4th instar nymphs, and 5-day-old adults (both females and males) for the predation assays. The 2nd instar represents the early instar stage with relatively weak predatory capacity, serving as a baseline for developmental comparison. The 4th instar represents the late instar stage, during which rapid growth and high nutritional demands may enhance predatory activity. The 5-day-old adults correspond to the peak reproductive stage, allowing assessment of predatory capacity during periods of elevated nutritional requirements.

To simulate the natural feeding behavior of *A. lucorum*, no starvation treatment was applied prior to the predation assays. All experiments were conducted under the same environmental conditions as insect rearing (26 ± 1 °C, 70 ± 5% RH, 14 L/10 D photoperiod). Each treatment was replicated five times.

(a) Predation on *H. armigera* eggs: Cotton gauze pieces (3 cm × 3 cm) containing *H. armigera* eggs (laid within 24 h) were prepared. Non-viable or misshapen eggs were removed under a stereomicroscope using an insect pin, and the number of eggs on each piece was recorded. Each gauze piece was placed in a glass tube (7.5 cm × 2 cm), and one *A. lucorum* individual (2nd instar nymph, 4th instar nymph, 5-day-old female, or 5-day-old male) was introduced using a soft brush. The tube opening was covered with an 80-mesh nylon net and secured with a rubber band to prevent escape. A moistened cotton ball was placed on the net to provide moisture. Prey densities tested were 5, 10, 20, 30, 40, and 50 eggs per tube, with five replicates per density. After 24 h, the number of consumed eggs was recorded under a stereomicroscope based on their shriveled, flattened appearance. Any eggs that had hatched within 24 h (which did not occur) or not shriveled were excluded from consumption counts.
(b) Predation on *A. gossypii* nymphs: Cotton leaves infested with *A. gossypii* nymphs were collected from the field and brought back to the laboratory. Under a stereomicroscope, healthy, unparasitized, and uninfected aphid nymphs (without distinction of instar to better reflect natural field conditions) were carefully selected using a fine brush and transferred onto cotton gauze pieces (3 cm × 3 cm). Each gauze piece containing the designated number of aphids was placed in a glass tubes (7.5 cm × 2 cm), and one *A. lucorum* individual (2nd instar nymph, 4th instar nymph, 5-day-old female, or 5-day-old male) was introduced into each tube. The tube opening was covered with an 80-mesh nylon net and secured with a rubber band to prevent escape, while a moistened cotton ball was placed on the net to provide moisture. Prey densities tested were 2, 4, 8, 12, 16, and 20 nymphs per tube, with five replicates per density. After 24 h, the predator was removed, and the number of consumed aphids (identified by their darkened, shriveled appearance) was recorded.
(c) Predation on *B. tabaci* nymphs: Cotton leaves infested with *B. tabaci* nymphs were collected from the field and brought back to the laboratory. The leaves were cut into leaf discs (3 cm × 3 cm). Under a stereomicroscope, excess nymphs, as well as unhealthy, parasitized, or infected individuals, were removed, leaving only healthy 4th instar nymphs to ensure uniformity of prey size. Each leaf disc was placed in a glass tube (7.5 cm × 2 cm), and one *A. lucorum* individual (2nd instar nymph, 4th instar nymph, 5-day-old female, or 5-day-old male) was introduced into each tube. The tube opening was covered with an 80-mesh nylon net and secured with a rubber band to prevent escape, while a moistened cotton ball was placed on the net to provide moisture. The prey densities tested were 2, 4, 8, 12, 16, and 20 nymphs per tube, with five replicates per density. After 24 h, the number of consumed nymphs (identified by their shriveled appearance) was recorded.

### 2.3. Field Detection of Predation by A. lucorum

#### 2.3.1. COI-Based Molecular Detection Method

(a) DNA template preparation: Genomic DNA was extracted from individual *A. lucorum* using a Blood/Cell/Tissue Genomic DNA Extraction Kit (Tiangen Biotech, Beijing, China) following the manufacturer’s instructions. The DNA extracts were stored at −20 °C until use. For PCR amplification, 1 µL of DNA solution was used as the template.
(b) Species-specific primers: Species-specific primers were designed based on mitochondrial COI gene sequences from *H. armigera* (GenBank accession No. AY264944) and *A. gossypii* (GenBank accession No. AY842502). Primers were synthesized by Sangon Biotech (Shanghai, China) and purified by PAGE.
  Primers for *H. armigera*:
  Forward primer MLCF: 5′-GGTGATCCTATTTTATATCAC-3′
  Reverse primer MLCR: 5′-GAGTATCAATATCTATACCAG-3′
  Amplicon length: 239 bp.
  Primers for *A. gossypii*:
  Forward primer MYF: 5′-TTCACATCAGCAACTATAATC-3′
  Reverse primer MYR: 5′-ACTACATAATAAGTGTCATGC-3′
  Amplicon length: 208 bp.
(c) PCR amplification: PCR reactions were performed in a thermal cycler (Bioer, Hangzhou, China). Each 25 µL reaction mixture contained 0.25 µL of EX Taq polymerase (TaKaRa, Dalian, China), 2.5 µL of 10× PCR buffer (supplied with the Taq polymerase), 2 µL of dNTPs (Tiangen Biotech, Beijing, China), 1 µL of DNA template, 0.5 µL of each forward and reverse primer, and sterile distilled water to a final volume of 25 µL. Each sample was run in duplicate to ensure reliability.
  The PCR cycling program was as follows: initial denaturation at 94 °C for 5 min; 36 cycles of 94 °C for 30 s, 55 °C for 30 s, and 72 °C for 1 min; final extension at 72 °C for 10 min.
  Positive controls (DNA from *H. armigera* eggs or *A. gossypii* nymphs) and negative controls (sterile water instead of DNA template, and DNA from *A. lucorum* that had not fed on *H. armigera* eggs or *A. gossypii*) were included.
(d) Amplicon detection: A 10 µL of each PCR product was electrophoresed on a 1.2% agarose gel containing 0.5 µg/mL GoldView nucleic acid stain (Solarbio, Beijing, China) at 180 V for 15 min. A DL2000 DNA molecular weight marker (TaKaRa, Dalian, China) was used as a reference. After electrophoresis, the gel was visualized and photographed under UV light using a gel imaging system (Tanon, Shanghai, China).

#### 2.3.2. Field Investigation and Sampling

Field surveys were conducted in cotton fields in Xinxiang City, Henan Province, Xiajin County, Shandong Province, and Langfang City, Hebei Province during the occurrence period of *A. lucorum* (from late June to early September) in 2009 and 2010. A diagonal five-point sampling method was used, with 10 cotton plants randomly examined at each sampling point, totaling 50 plants per survey site. Surveys were carried out every 3–5 days, and the number of *A. lucorum adults*, *H. armigera* eggs, and *A. gossypii* nymphs per 50 plants were recorded.

In addition, adult *A. lucorum* were collected during three key periods: the early occurrence stage (late June to early July), the peak occurrence stage (mid-July to early August), and the late occurrence stage (mid-August to early September). Adults were collected using insect nets or aspirators, with as many individuals as possible collected. Collected specimens were placed separately into 2 mL centrifuge tubes containing 100% ethanol, transported to the laboratory within 1 h, then stored at −20 °C or −80 °C for subsequent DNA extraction and PCR analysis.

### 2.4. Statistical Analysis

All data were analyzed using SAS 9.4 (SAS Institute Inc., Cary, NC, USA), and functional response curve fitting was conducted using R 4.1.3 (R Core Team, Vienna, Austria). A significance level of *p* < 0.05 was used for all statistical tests. All data are presented as mean ± standard error of the mean (SE).

One-way ANOVA with Tukey’s HSD test (SAS 9.4) was used to compare prey consumption among different developmental stages (2nd instar nymphs, 4th instar nymphs, 5-day-old females, and 5-day-old males) of *A. lucorum* at each prey density for each prey species (*H. armigera* eggs, *A. gossypii* nymphs, and *B. tabaci* nymphs). Five replicates were conducted per density. Normality and homogeneity of variance were verified using Shapiro–Wilk and Levene’s tests, respectively. 

To determine the functional response type, logistic regression was performed on the proportion of prey consumed (*Na*/*N*) as a function of initial prey density (*N*). The model was fitted using a binomial distribution with a logit link function, weighted by initial prey density:

*Na*/*N* = exp (*P*_0_ + *P*_1_ *N* + *P*_2_ *N*_2_ + *P*_3_ *N*_3_)/1 + exp (*P*_0_ + *P*_1_ *N* + *P*_2_ *N*_2_ + *P*_3_ *N*_3_) (1)

The signs and significance of *P*_1_ and *P*_2_ were used to determine the functional response type. A Type II response is indicated by a significantly negative linear coefficient (*P*_1_ < 0, *p* < 0.05), whereas a Type III response is indicated by a significantly positive linear coefficient and a significantly negative quadratic coefficient (*P*_2_ < 0, *p* < 0.05). For combinations where *P*_1_ was not significant, the functional response type was further evaluated using Holling’s disc equation, with *R*^2^ was used as a complementary criterion [31,32].

The predation data conformed to the Holling Type II disc equation [33]:

*Na* = *aTN*/(1 + *aThN*) (2)

where *Na* is the number of preys consumed, *N* is the initial prey density, *a* is the attack rate, *T* is the experimental time (*T* = 1 d), and *Th* is the handling time. Maximum daily prey consumption was calculated as 1/*Th*, and predation capacity as *a*/*Th*. The equation was linearized as

1/*Na* = 1/*aT* ×1/*N* +*Th*/*T* (3)

For the population dynamics data, differences in pest densities (*A. lucorum* adults, *H. armigera* eggs, and *A. gossypii* nymphs) among sampling dates were analyzed separately for each location and year using repeated measures ANOVA, followed by Tukey’s HSD test for multiple comparisons. Normality assumptions were tested using the Shapiro–Wilk test.

For the molecular detection data, the positive detection rates of *A. gossypii* DNA were analyzed separately for each region and year using repeated measures ANOVA. Tukey’s HSD test was used for multiple comparisons. Normality assumptions were tested using the Shapiro–Wilk test. Detection rates of *H. armigera* eggs were zero across all samples and were therefore excluded from statistical analysis.

## 3. Results

### 3.1. Functional Response Type

Logistic regression analysis was employed to determine the functional response types of different developmental stages of *A. lucorum* to three prey species. The proportion of prey consumed (*Na*/*N*) was modeled as a function of initial prey density (*N*), and the signs and significance of the linear coefficient (*P*_1_) and quadratic coefficient (*P*_2_) were used to identify the functional response type. Across all predator–prey combinations, the linear coefficient (*P*_1_) was consistently negative (Table 1), indicating that the proportion of prey consumed decreased with increasing prey density—a characteristic pattern of a Type II functional response.

Among the 12 predator–prey combinations, only the combination of *H. armigera* eggs with 5-day-old female adults yielded a statistically significant linear coefficient (*P*_1_ = −0.6754, *p* = 0.0004). For all other combinations, the linear coefficients were negative but not significant (*p* > 0.05). The quadratic coefficients (*P*_2_) were positive across all combinations, which does not satisfy the criteria for a Type III functional response (*P*_1_ > 0 and *P*_2_ < 0). To further validate the functional response type, nonlinear regression was performed using Holling’s disc equation. The Holling Type II model exhibited excellent goodness-of-fit for all predator–prey combinations, with *R*^2^ values ranging from 0.9029 to 0.9965 (Table 1). Taken together, the results from both logistic regression and Holling Type II model fitting confirm that all developmental stages of *A. lucorum* (2nd instar nymphs, 4th instar nymphs, 5-day-old females, and 5-day-old males) exhibit a Type II functional response when preying on *H. armigera* eggs, *A. gossypii* nymphs, and *B. tabaci* nymphs.

### 3.2. Predation Rates of A. lucorum on Three Prey Types

**Figure 1:**
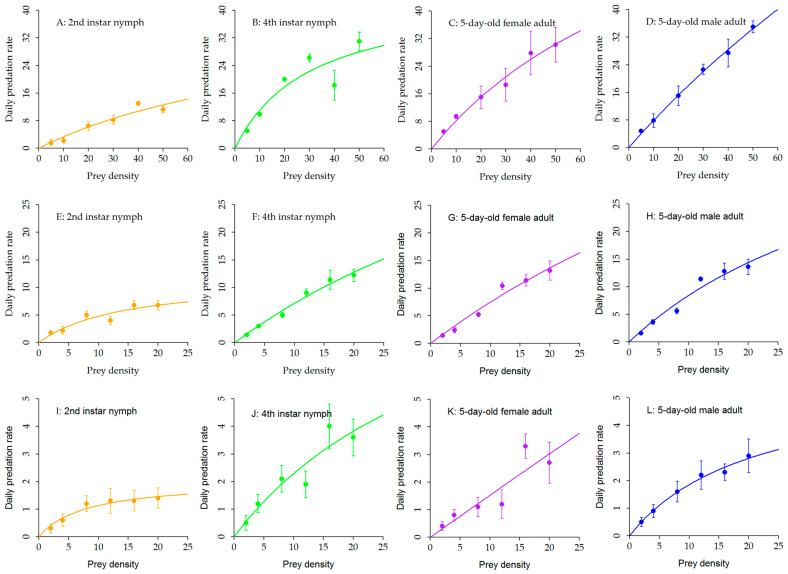
Predation responses of *Apolygus lucorum* at different developmental stages to three prey species: *Helicoverpa armigera* eggs (**A**–**D**), *Aphis gossypii* nymphs (**E**–**H**), and *Bemisia tabaci* nymphs (**I**–**L**). Each subfigure represents a specific developmental stage: 2nd instar nymphs (**A**,**E**,**I**), 4th instar nymphs (**B**,**F**,**J**), 5-day-old female adults (**C**,**G**,**K**), and 5-day-old male adults (**D**,**H**,**L**). Data are presented as mean daily predation rate ± SE (*n* = 5 per prey density). Curves were fitted using the Holling type II disc equation. Significant differences in prey consumption among developmental stages were analyzed using one-way ANOVA followed by Tukey’s HSD test. Colors in the figure represent different developmental stages: orange, 2nd instar nymph; green, 4th instar nymph; purple, 5-day-old female; blue, 5-day-old male.

The predation rates of *A. lucorum* at different developmental stages increased with prey density in a decelerating manner and tended to approach saturation at higher densities (Figure 1). The functional response parameters, including attack rate (*a*), handling time (*Th*), maximum daily consumption (1/*Th*), and predation capacity (*a*/*Th*), varied considerably among prey types and predator developmental stages (Table 2).

For *H. armigera* eggs, 2nd instar nymphs reached their maximum consumption (13.00 eggs) at a density of 40 eggs, with no significant difference compared to consumption at 50 eggs (11.20 eggs, *p* > 0.05). In contrast, the maximum predation rates of 4th instar nymphs, 5-day-old females, and 5-day-old males all occurred at the highest egg density (50 eggs), with values of 31.00, 30.20, and 35.00 eggs, respectively. Across all densities, 2nd instar nymphs consistently exhibited the lowest predation levels (Figure 1A–D). Parameter estimates further supported these observations: 4th instar nymphs and 5-day-old males had the highest predation capacities (*a*/*Th* = 75.71 and 73.13, respectively), while 2nd instar nymphs showed the lowest (13.08), consistent with their lower consumption across densities (Table 2).

On *A. gossypii* nymphs, the predation rate of 2nd instar nymphs fluctuated, peaking at densities of 16 and 20 nymphs (both 6.80 individuals). Consumption at a density of 8 nymphs (5.00 individuals) was higher than at densities of 2, 4, and 12 nymphs. For 4th instar nymphs, 5-day-old females, and 5-day-old males, predation rates increased steadily with aphid density, reaching maxima at 20 nymphs (12.20, 13.20, and 13.60 nymphs, respectively). Across all densities, 5-day-old males consistently consumed more than 5-day-old females. Fourth instar nymphs generally outperformed 2nd instar nymphs, except at the lowest density (2 nymphs) where consumption was lower, and at 8 nymphs where they were comparable (Figure 1E–H). The functional response parameters highlighted these stage-specific differences: 5-day-old males exhibited the highest predation capacity (*a*/*Th* = 679.42) and theoretical maximum daily consumption (833.33 aphids), followed by 5-day-old females (273.24 and 400.00 aphids), while 2nd instar nymphs had the lowest values (8.52 and 7.97 aphids) (Table 2).

In assays with *B. tabaci* nymphs, predation rates generally increased with prey density. Fourth instar nymphs exhibited the strongest functional response, displaying a rapid increase in consumption across the density range—particularly a sharp rise from 12 to 16 nymphs (from 1.90 to 4.00 nymphs)—and reaching the highest maximum consumption (3.60 nymphs) at a density of 20 nymphs. Five-day-old females and males showed more gradual increases without clear inflection points, attaining maxima of 2.70 and 2.90 nymphs, respectively, also at the highest density. In contrast, 2nd instar nymphs reached saturation earlier, with consumption stabilizing at 1.30–1.40 nymphs after a density of 12 nymphs, indicating a lower predation ceiling and earlier saturation. At identical prey densities, 4th instar nymphs consistently outperformed other stages, while 2nd instar nymphs exhibited the lowest predation across the entire density range (Figure 1I–L). Parameter estimates showed that predation capacity for *B. tabaci* was substantially lower than for the other two prey species. Fourth instar nymphs had the highest *a*/*Th* (4.48) and maximum consumption (16.75 nymphs), whereas 2nd instar nymphs again showed the lowest values (0.58 and 3.47 nymphs) (Table 2).

Overall, the functional responses of *A. lucorum* at different developmental stages to all three prey species conformed to the Holling type II model. Predation capacity and maximum daily consumption increased with developmental stage, with 2nd instar nymphs consistently showing the lowest efficiency and 4th instar nymphs and adults exhibiting significantly higher predatory performance. Notably, 5-day-old males consistently outperformed females across most prey types and densities, suggesting a sex-based difference in foraging strategy, particularly evident in the high predation capacity observed on *A. gossypii*.

### 3.3. Detection of Prey Predation by A. lucorum in Cotton Fields

The predatory interactions of *A. lucorum* with *H. armigera* eggs and *A. gossypii* were evaluated using COI molecular markers on individuals sampled from Bt cotton fields. Analysis indicated no positive predation signals for *H. armigera* eggs in any tested samples from 2009 and 2010. In contrast, predation on *A. gossypii* by *A. lucorum* was observed, though with pronounced regional variation. In Langfang, positive detections of *A. gossypii* predation were recorded consistently across both study years. In 2009, 14 positive samples were identified (5 collected on 15 July and 8 on 18 July), with an average detection rate of 7.04% ± 3.73. In 2010, 8 positive samples were collected between 25 July and 2 August, with an average detection rate of 3.70% ± 2.47. In Xiajin, three positive samples obtained on 27 July 2009, corresponded to an average detection rate of 1.22% ± 1.26, whereas no positive signals were detected in 2010. In Xinxiang, no *A. gossypii* DNA was detected in either year, resulting in a 0% positive detection rate across both sampling periods (Table 3).

**Figure 2:**
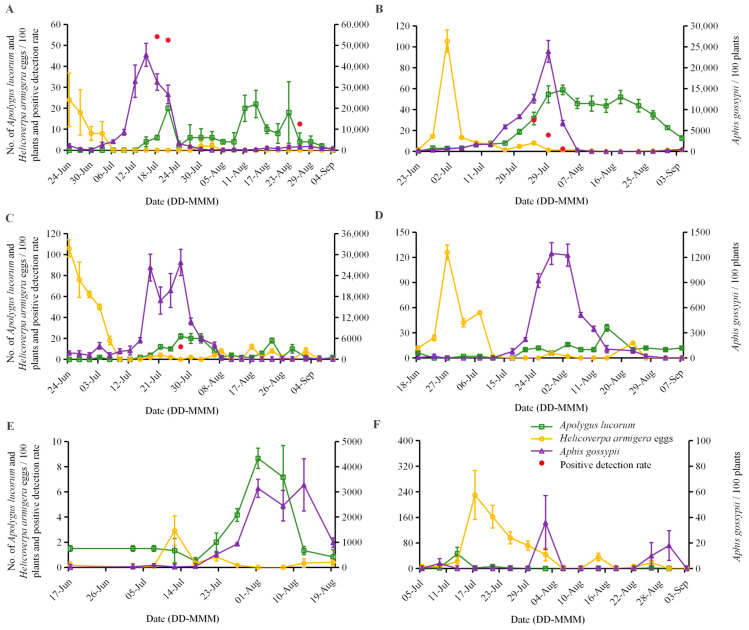
Population dynamics of *Apolygus lucorum*, *Helicoverpa armigera* eggs, and *Aphis gossypii* in cotton fields, and the positive detection rate of *A. gossypii* DNA in field-collected *A. lucorum* individuals. Data are shown for Langfang (**A**,**B**), Xiajin (**C**,**D**), and Xinxiang (**E**,**F**) in 2009 (**A**,**C**,**E**) and 2010 (**B**,**D**,**F**). Left *y*-axis indicates the number of *A. lucorum* adults and *H. armigera* eggs per 100 plants, as well as the positive detection rate (%) of *A. gossypii* DNA. Right *y*-axis indicates the number of *A. gossypii* nymphs per 100 plants. Data are presented as mean ± SE (*n* = 5 sampling points per date).

To elucidate the relationship between molecular detection results and actual field ecology, positive detection rates were systematically analyzed alongside the population dynamics of *A. lucorum*, *H. armigera* eggs, and *A. gossypii* during sampling. Positive detections for *A. gossypii* consistently coincided with peaks in aphid populations, which also corresponded to periods of higher *A. lucorum* density. In Langfang, positive detections in 2009 corresponded to sampling dates on 15 July, 18 July, and 26 August, with *A. gossypii* densities of 45,460.00 ± 5480.09, 32,500.00 ± 3904.92, and 1522.00 ± 715.78 individuals per 100 plants, respectively (Figure 2A). In 2010, positive detections were recorded on 25 July, 29 July, and 2 August, accompanied by *A. gossypii* densities of 12,596.00 ± 1018.56, 23,917.00 ± 2589.97, and 6792.00 ± 657.56 individuals per 100 plants, respectively (Figure 2B). In Xiajin, all three positive reaction samples in 2009 were collected on July 27, when *A. gossypii* density was 27,760 ± 3757.31 individuals per 100 plants. This date coincided with both the peak occurrence of *A. gossypii* and the population peak of *A. lucorum* (Figure 2C). In 2010, despite sufficient sampling effort, the *A. gossypii* population remained low, with a peak density of only 1246.00 ± 129.38 individuals per 100 plants, and no positive detections were obtained (Figure 2D). In Xinxiang, although the occurrence periods of *A. gossypii* and *A. lucorum* broadly overlapped in 2009, the population density of *A. lucorum* was low and the sample size was limited, resulting in no positive reactions (Figure 2E,F). In 2010, both *A. gossypiis* and *A. lucorum* occurred only sporadically, and again no positive reactions were detected (Figure 2F).

As shown in Figure 2, *H. armigera* egg populations in Bt cotton fields remained at consistently low densities throughout the study period. The highest recorded egg count across all three regions and both years was only 230.00 ± 76.85 eggs per 100 plants. During peaks in *H. armigera* egg occurrence, *A. lucorum* was consistently in its early occurrence period in the cotton fields, characterized by low overall population abundance and limited sample availability for molecular analysis. Consequently, no target *H. armigera* DNA fragments were amplified from any tested *A. lucorum* individuals.

Analysis based on COI molecular markers indicated that under natural field conditions, *A. lucorum* did not exhibit significant predation on *H. armigera* eggs, whereas it showed detectable predation on *A. gossypii*. Moreover, predation rates varied markedly among geographical regions, with the most substantial predatory activity observed in Langfang. These findings suggest that the feeding behavior of *A. lucorum* in agroecosystems may be shaped by both spatiotemporal dynamics and prey selectivity.

## 4. Discussion

This study systematically evaluated the predatory functional responses of *A*. *lucorum* at different developmental stages (2nd instar, 4th instar nymphs, and 5-day-old male and female adults) to *H*. *armigera* eggs, *A*. *gossypii* nymphs, and *B*. *tabaci* nymphs. The results indicated that the predatory behavior of *A. lucorum* towards all three prey types conformed to the Holling type II functional response model, with predation initially increasing and then stabilizing as prey density rose. This pattern aligns with observations of *N*. *tenuis* predation of *B. tabaci*, *Calvia muiri* (Coleoptera: Coccinellidae) on *Panaphis juglandis* (Hemiptera: Aphididae), and *Arma chinensis* (Hemiptera: Pentatomidae) on *Spodoptera frugiperda* (*Lepidoptera*: *Noctuidae*) [34,35,36,37], suggesting that such functional responses are common among predatory insects.

The study further revealed significant influences of developmental stage and sex on predation capacity. When preying on *H. armigera* eggs, 5-day-old males exhibited the highest predation potential, with a theoretical maximum of 73.53 eggs, followed by 4th instar nymphs (68.97 eggs), while 2nd instar nymphs showed the weakest predation capacity (41.84 eggs). For *A. gossypii* nymphs, predation differences among stages were more pronounced: 5-day-old males consumed the most (833.33 individuals), significantly higher than 5-day-old females (400.00 individuals), with 2nd instar nymphs consuming the least (7.97 individuals). When preying on *B. tabaci* nymphs, 4th instar nymphs displayed the strongest predation capacity (16.75 individuals), followed by 5-day-old males (5.74 individuals), while 5-day-old females consumed the least (3.42 individuals). Overall, older nymphs and adults showed a preference for *A. gossypii*, whereas younger nymphs exhibited a relative preference for *H. armigera* eggs, indicating that the predatory strategy of *A. lucorum* is significantly influenced by both its developmental stage and prey type. Similar phenomena have been reported in other predatory insects. For instance, when *S. frugiperda* and *Rhopalosiphum padi* (Hemiptera: Aphididae) coexist, *Harmonia axyridis* (Coleoptera: Coccinellidae) preferentially predates the latter [38]. Further, Cai et al. [39] found that 2nd instar larvae and female adults of *H. axyridis* exhibited higher predation rates on *Aphis spiraecola* (Hemiptera: Aphididae) than on *Semiaphis heraclei* (Hemiptera: Aphididae), while 3rd instar larvae and male adults showed a stronger preference for *S. heraclei*. These findings collectively highlight the widespread interaction between developmental stage and prey type in predatory insect behavior.

From a developmental perspective, 4th instar nymphs exhibited greater predation capacity than 2nd instar nymphs, indicating enhanced predatory ability with advancing developmental stage. This trend aligns with findings on *N. tenuis*, whose predation on *B. tabaci* and *Thrips palmi* (Thysanoptera: Thripidae) increases with nymphal stage [40]. Regarding sex differences, males consumed more of all prey types than females. This is consistent with observations of *Orius albidipennis* (Hemiptera: Anthocoridae), where males displayed significantly higher attack rates on *Aphis fabae* (Hemiptera: Aphididae) than females [41]. This may be related to adaptive behavioral strategies developed by males in mating competition and resource acquisition, with stronger predation capacity likely conferring a competitive advantage within the population [42].

Notably, the predation characteristics of *A. lucorum,* which are significantly regulated by developmental stage, prey type, and density, are also observed in other mirid species that are primarily phytophagous but facultatively predatory. For example, *A*. *suturalis* tends to feed on a mix of plant and animal materials, and its predation on *A*. *ipsilon* and *S*. *exigua* follows the Holling type II functional response [16,43]. Similarly, *Lygus pratensis* (Hemiptera: Miridae) stages exhibit enhanced predation on *A. gossypii* with increased aphid density and nymphal stage, which was further confirmed by field cage experiments [44]. These species demonstrate strong dependence of predatory behavior on prey population density and availability, suggesting that facultative predation may represent an important ecological plasticity for adaptation to agricultural ecosystems.

Although laboratory experiments confirmed the predation capacity of *A. lucorum*, molecular detection and field population dynamics analyses jointly indicate significant limitations to its actual predatory role under natural conditions. Specifically, *A. lucorum* consistently failed to exhibit effective predation on *H. armigera* eggs in the field, while its predation on *A. gossypii* showed marked regional variation and was strictly concentrated during peak aphid population periods. These contrasting results, on one hand, support the laboratory findings of highest predation capacity on *A. gossypii*, indicating clear prey preference and population dependence; on the other hand, they reveal that predation on *H. armigera* eggs is largely unfeasible in the field, as it is constrained by extremely low prey density, high search costs, and phenological asynchrony. These findings underscore that the predatory behavior of *A. lucorum* is not only influenced by prey type but also closely linked to the spatiotemporal dynamics and population density of prey in the field, highlighting its resource dependence and ecological plasticity as a facultative predator. Consistent with this view, field predation events were detected only during periods of high prey density and were absent when prey populations were low, indicating that the laboratory-derived maximum consumption rates (e.g., 833 aphids/day) are rarely realized under natural conditions due to ecological constraints such as low prey availability, phenological asynchrony, and the pest’s dominant phytophagous behavior.

The transition between phytophagy and zoophagy in *A. lucorum* appears to be governed by a combination of physiological demands and ecological context. Laboratory results showed that older nymphs and adult males exhibited the highest predation rates, suggesting that periods of rapid growth or reproduction may increase the demand for protein-rich animal prey—a pattern consistent with observations in other omnivorous mirids such as *N*. *tenuis* and *M*. *pygmaeus* [45,46]. In contrast, under field conditions, such predatory activity becomes evident only when prey are abundant, further supporting the view that zoophagy in this species is predominantly opportunistic rather than obligate. Moreover, its well-documented host-switching behavior—moving among jujube, cotton, and other crops in response to plant phenology—indicates that plant resources remain the primary driver of its population dynamics [47,48]. Thus, while facultative predation may provide nutritional benefits that enhance fitness under specific conditions, it does not alter its fundamental status as a phytophagous pest.

Analyzed in conjunction with its seasonal host-switching behavior, *A. lucorum* tends to shift towards flowering plants during host transitions [49,50]. When cotton growth enters peak flowering in July, *A. lucorum* migrates into cotton fields and initiates damage. This migration pattern results in spatiotemporally limited predatory behavior in the field, occurring sporadically only under specific conditions—such as during cotton peak flowering and when high densities of specific prey (e.g., *A. gossypii*) are present. Although *A. lucorum* may transiently regulate populations of minor pests like *A. gossypii* within food webs, its phytophagous damage remains dominant. Predation is merely a supplementary component of its nutritional strategy and does not alter its ecological role as a major pest. Therefore, while the laboratory findings confirm the facultative predatory capacity of *A. lucorum*, the field evidence demonstrates that this capacity does not translate into reliable biological control under natural conditions. Within an IPM framework, management strategies should continue to prioritize the suppression of *A. lucorum* as a direct pest, particularly during its peak occurrence periods and host-switching phases. The predatory behavior documented in this study should not be interpreted as a justification for conserving or augmenting *A. lucorum* populations, as such approaches would risk exacerbating its phytophagous damage. Instead, these findings contribute to a more nuanced ecological understanding of a dominant pest species, informing risk assessment and supporting the development of multi-trophic management strategies that account for complex species interactions within agricultural ecosystems.

## 5. Conclusions

By integrating laboratory functional response assays, molecular detection, and field ecological surveys, this study systematically clarifies the ecological characteristics and realizes function of the facultative predation behavior exhibited by the primarily phytophagous pest *A*. *lucorum*. The results indicate that predation by *A. lucorum* on *H*. *armigera* eggs, *A*. *gossypii* nymphs, and *B*. *tabaci* nymphs conforms to the Holling type II functional response model. Predation capacity was significantly affected by the predator’s developmental stage, sex, and prey type, with older nymphs and male adults demonstrating the highest predation potential on *A. gossypii* nymphs. However, actual predation impact in the field was strongly limited by prey population density, spatiotemporal availability, and regional ecological conditions. No effective predation on *H. armigera* eggs was documented, while predation on *A. gossypii* displayed clear regional heterogeneity. The acquisition of animal nutrition through facultative predation may improve the population fitness and damage potential of *A. lucorum* under resource-limited conditions, thereby complicating integrated pest management (IPM) approaches. Therefore, within an IPM framework, it is recommended to implement a management system based on dynamic monitoring and ecological risk assessment. This approach supports region-specific and temporally targeted precision control strategies, which would help avoid unintended enhancement of the pest’s ecological competitiveness during management interventions.

## Figures and Tables

**Table 1:** Logistic regression parameters and Holling Type II model goodness-of-fit for the functional responses of different developmental stages of *Apolygus lucorum* to three prey species.

Prey	Developmental Stage	Parameter	Estimate	Standard Error	*p*-Value	*R* ^2^
*Helicoverpa armigera* eggs	2nd instar nymph	Linear (*P*_1_)	−0.0648	0.0940	0.4906	0.9199
		Quadratic (*P*_2_)	0.0034	0.0035	0.3415	
	4th instar nymph	Linear (*P*_1_)	−0.6235	0.5008	0.2132	0.9736
		Quadratic (*P*_2_)	0.0160	0.0147	0.2743	
	5-day-old female adult	Linear (*P*_1_)	−0.6754	0.1924	0.0004	0.9784
		Quadratic (*P*_2_)	0.0198	0.0061	0.0012	
	5-day-old male adult	Linear (*P*_1_)	−0.1182	0.1104	0.2844	0.9965
		Quadratic (*P*_2_)	0.0029	0.0040	0.4744	
*Aphis gossypii* nymph	2nd instar nymph	Linear (*P*_1_)	−0.3019	0.3630	0.4056	0.9655
		Quadratic (*P*_2_)	0.0181	0.0336	0.5894	
	4th instar nymph	Linear (*P*_1_)	−0.3196	0.3735	0.3922	0.9802
		Quadratic (*P*_2_)	0.0356	0.0350	0.3086	
	5-day-old female adult	Linear (*P*_1_)	−0.1879	0.3728	0.6142	0.9637
		Quadratic (*P*_2_)	0.0350	0.0357	0.3246	
	5-day-old male adult	Linear (*P*_1_)	−0.3590	0.4513	0.4263	0.9573
		Quadratic (*P*_2_)	0.0451	0.0422	0.2858	
*Besimia tabaci* nymph	2nd instar nymph	Linear (*P*_1_)	−0.1597	0.3687	0.6649	0.9141
		Quadratic (*P*_2_)	0.0123	0.0359	0.7315	
	4th instar nymph	Linear (*P*_1_)	−0.1005	0.2703	0.7100	0.9540
		Quadratic (*P*_2_)	0.0108	0.0257	0.6750	
	5-day-old female adult	Linear (*P*_1_)	−0.2890	0.2994	0.3344	0.9029
		Quadratic (*P*_2_)	0.0331	0.0282	0.2404	
	5-day-old male adult	Linear (*P*_1_)	−0.0530	0.2984	0.8593	0.9556
		Quadratic (*P*_2_)	0.0025	0.0288	0.9320	

**Table 2:** Functional response parameters of *Apolygus lucorum* at different developmental stages preying on eggs of *Helicoverpa armigera*, nymphs of *Aphis gossypii*, and nymphs of *Bemisia tabaci*.

Prey	Developmental Stage	Functional Response	*p*	*a*	*Th* (d)	Maximum Consumption	*a*/*Th*
*Helicoverpa armigera* eggs	2nd instar nymph	*Na* = *N*/(3.1989 + 0.0239 *N*)	0.0010	0.3126	0.0239	41.84	13.08
	4th instar nymph	*Na* = *N*/(0.9109 + 0.0145 *N*)	0.0002	1.0978	0.0145	68.97	75.71
	5-day-old female adult	*Na* = *N*/(0.9113 + 0.0176 *N*)	<0.0001	1.0973	0.0176	56.82	62.35
	5-day-old male adult	*Na* = *N*/(1.0055 + 0.0136 *N*)	<0.0001	0.9945	0.0136	73.53	73.13
*Aphis gossypii* nymph	2nd instar nymph	*Na* = *N*/(0.9358 + 0.1254 *N*)	0.0048	1.0686	0.1254	7.97	8.52
	4th instar nymph	*Na* = *N*/(1.4069 + 0.0037 *N*)	<0.0001	0.7108	0.0037	270.27	192.11
	5-day-old female adult	*Na* = *N*/(1.4640 + 0.0025 *N*)	<0.0001	0.6831	0.0025	400.00	273.24
	5-day-old male adult	*Na* = *N*/(1.2265 + 0.0012 *N*)	<0.0001	0.8153	0.0012	833.33	679.42
*Besimia tabaci* nymph	2nd instar nymph	*Na* = *N*/(5.9340 + 0.2886 *N*)	<0.0001	0.1685	0.2886	3.47	0.58
	4th instar nymph	*Na* = *N*/(3.7406 + 0.0597 *N*)	0.0003	0.2673	0.0597	16.75	4.48
	5-day-old female adult	*Na* = *N*/(5.2068 + 0.2926 *N*)	0.0200	0.1921	0.2926	3.42	0.66
	5-day-old male adult	*Na* = *N*/(3.6654 + 0.1742 *N*)	<0.0001	0.2728	0.1742	5.74	1.57

*p* values indicate the significance level of the Holling type II model fitting. *a* is the attack rate, and *Th* is the handling time (d). Maximum daily prey consumption was calculated as 1/*Th*, and predation capacity was calculated as *a*/*Th*. The total experimental time was *T* = 1 d. All treatments conformed to the Holling type II model based on functional response curve fitting.

**Table 3:** Positive detection rates of *Helicoverpa armigera* eggs and *Aphis gossypii* in cotton fields from three regions during 2009–2010.

Year	Area	Total No. of Detections	Positive Detection Rate of *Helicoverpa armigera* Eggs	Positive Detection Rate of *Aphis gossypii*
2009	Langfang	126	0.00	7.04 ± 3.73 a
	Xiajin	141	0.00	1.22 ± 1.26 a
	Xinxiang	42	0.00	0.00 ± 2.47 a
2010	Langfang	135	0.00	3.70 ± 2.47 a
	Xiajin	114	0.00	0.00 ± 1.23 a
	Xinxiang	41	0.00	0.00 ± 1.23 a

Data for positive detection rate are presented as means ± SE. Means in the same column followed by different letters are significantly different for each year (*p* < 0.05) (one-way ANOVA, Tukey’s HSD).

## Data Availability

The original contributions presented in this study are included in the article. Further inquiries can be directed to the corresponding author.

## References

[1] Chen H., Su H.H., Zhang S., Jing T.X., Liu Z., Yang Y.Z. (2022). Transcriptomic and metabolomic responses in cotton plant to *Apolygus lucorum* infestation. Insects.

[2] Tan W., Yin Q., Zhao H., Wang M.Y., Sun X., Cao H., Wang D.Y., Li Q.L. (2025). Disruption of chlorophyll metabolism and photosynthetic efficiency in winter jujube (*Ziziphus jujuba*) induced by *Apolygus lucorum* infestation. Front. Plant. Sci..

[3] Yao H., Gao S.H., Sun T.H., Zhou G.N., Lu C.K., Gao B.J., Chen W.S., Liang Y.M. (2024). Transcriptomic analysis of the defense response in “Cabernet Sauvignon” grape leaf induced by *Apolygus lucorum* feeding. Plant Direct.

[4] Wang X.L., Su H., Wang J., Li G.P., Feng H.Q., Zhang J.Y. (2023). Monitoring of insecticide resistance for *Apolygus lucorum* populations in the apple orchard in China. Crop Prot..

[5] Wu J., Cao Y., Teng D., Shan S., Geng T., Huang X.Z., Zhang Y.J. (2024). Volatiles of different resistant cotton varieties mediate the host preference of Mirid bug *Apolygus lucorum*. Front. Plant Sci..

[6] Wang K.T., Yang L., Pang Y.F., Lu Y.H. (2024). Seasonal host transfer patterns of *Apolygus lucorum* in southern Xinjiang agroecosystem. China Plant Prot..

[7] Adel K., An X.h., Shan S., Pang X.q., Li Y., Fu X.W., Zhang Y.J. (2022). The microRNAs in the antennae of *Apolygus lucorum* (Hemiptera: Miridae): Expression properties and targets prediction. Genomics.

[8] Pan H.S., Xiu C.L., Liu B., Lu Y.H. (2019). Plant stalks as oviposition traps for *Apolygus lucorum* (Hemiptera: Miridae) under field condition. Int. J. Pest Manag..

[9] Jiang R.Z., Xu G.C., Qiao H., Zhao J., Xiao L.B., Salzman K.Z., Xu D.J., Shen J., Hao D.J., Yan S. (2025). Nanodelivery improves insecticidal activity of Hexaflumuron against *Apolygus lucorum* by inhibiting the synthesis of insect cuticle protein. J. Agric. Food Chem..

[10] Liu H.W., Sun X.J., Shi Z., An X.K., Khashaveh A., Li Y., Gu S.H., Zhang Y.J. (2022). Identification and functional analysis of odorant-binding proteins provide new control strategies for *Apolygus lucorum*. Int. J. Biol. Macromol..

[11] Tian Y.Y., Wang H.Y., Hou J., Zhang L.X., Zhang Z.Q. (2019). Occurrence and Distribution of *Apolygus lucorum* on Weed Hosts and Tea Plants in Tea Plantation Ecosystems. Insects.

[12] Wang L.L., Lu Y.H., Wu K.M. (2010). The method of COΙ marker for detecting predation of *Apolygus lucorum* on Helicoverpa armigera eggs. Entomol. Knowl..

[13] Li W.J., Wyckhuys G.A.K., Wu K.M. (2016). Does feeding behavior of a zoophytophagous mirid differ between host plant and insect prey items?. Arthropod-Plant Interact..

[14] Cassi S.M., Dumont F., Provost C., Lucas E. (2024). Enhancing Biological Control Efficacy: Insights into the Feeding Behavior and Fitness of the Omnivorous Pest *Lygus lineolaris*. Insects.

[15] Dumont F., Cassi S.M., Provost C. (2025). Genetic variation in zoophagy and dietary shift in the phytozoophagous tarnished plant bug, *Lygus lineolaris*. Entomol. Exp. Appl..

[16] Feng L.K., Xu Q., Shu M., Wang P.L. (2016). Predation functional respones of *Adelphocoris lineolatus* Goeze adults to the Leptinotarsa decemlineata Say low instar larvae. J. Shihezi Univ. (Nat. Sci.).

[17] Li W.J., Lu Y.H., Gao X.W., Wu K.M. (2012). Predation of *Adelphocoris suturalis* on immature stages of *Agrotis ypsilon* and *Spodoptera exigua*. J. Appl. Entomol..

[18] Coll M., Guershon M. (2002). Omnivory in terrestrial artropods: Mixing plant and prey diets. Annu. Rev. Entomol..

[19] Gillespie R.D., Mcgregor R.R. (2000). The functions of plant feeding in the omnivorous predator *Dicyphus hesperus*: Water places limits on predation. Ecol. Entomol..

[20] Lundgren J.G. (2009). Relationships of Natural Enemies and Non-Prey Foods.

[21] Symondson W.O.C., Sunderland K.D., Greenstone M.H. (2002). Can generalist predators be effective biocontrol agents?. Annu. Rev. Entomol..

[22] Fussmann F.G., Heber G. (2002). Food web complexity and chaotic population dynamics. Ecol. Lett..

[23] Wise D.H. (2006). Cannibalism, food limitation, intraspecific competition, and the regulation of spider populations. Annu. Rev. Entomol..

[24] Hashimoto N., Yano E., Kandori I. (2025). Feeding preference of *Nesidiocoris tenuis* (Hemiptera: Miridae) between *Bemisia tabaci* (Hemiptera: Aleyrodidae) and *Thrips palmi* (Thysanoptera: Thripidae). Appl. Entomol. Zool..

[25] Gavkare O., Sharma L.P., Sanchez A.J., Shah M.A. (2017). Functional response of *Nesidiocoris tenuis* (Hemiptera: Miridae) to the two-spotted spider mite, *Tetranychus urticae*. Biocontrol Sci. Technol..

[26] Leman A., Ingegno B.L., Tavella L., Arne J., Messelink G.J. (2020). The omnivorous predator *Macrolophus pygmaeus*, a good candidate for the control of both greenhouse whitefly and poinsettia thrips on gerbera plants. Insect Sci..

[27] Lu Y.H., Wu K.M., Jiang Y., Xia B., Li P., Feng H.Q., Wyckhuys K.A.G., Guo Y.Y. (2010). Mirid Bug Outbreaks in Multiple Crops Correlated with Wide-Scale Adoption of Bt Cotton in China. Science.

[28] Zhang T., Mei X.D., Zhang X.F., Lu Y.H., Ning J., Wu K.M. (2020). Identification and field evaluation of the sex pheromone of *Apolygus lucorum* (Hemiptera: Miridae) in China. Pest Manag. Sci..

[29] Lu Y.H., Wu K.M., Cai X.M., Liu Y.Q. (2008). A rearing method for mirids using the green beans, *Phaseolus vulgaris* in the laboratory. J. Plant Prot..

[30] Liang G., Tan W.J., Guo Y.Y. (1999). Improvement in artificial rearing techniques for *Helicoverpa armigera*. Plant Prot..

[31] Juliano S., Scheiner S.M., Gurevitch J. (2001). Nonlinear curve fitting: Predation and functional response curves. Design and Analysis of Ecological Experiments.

[32] Bosomtwe A., Opit G., Giles K., Kard B., Goad C. (2025). Functional Responses of the Warehouse Pirate Bug *Xylocoris flavipes* (Reuter) (Hemiptera: Anthocoridae) on a Diet of *Liposcelis decolor* (Pearman) (Psocodea: Liposcelididae). Insects.

[33] Holling C.S. (1959). Some characteristics of simple types of predation and parasitism. Can. Entomol..

[34] Xiao Q.L., Zhang Y.T., Yang C.Y., Pan H.D., Li C.Y., Xie H.D., Peng Y., Jiang W.H., Ren G.W., Xu P.J. (2025). Predatory functional response of *Nesidiocoris tenuis* to *Bemisia tabaci*, *Myzus persicae*, and *Spodoptera frugiperda*. Chin. J. Biol. Control.

[35] Jiang H., Yang X.X., Li G.L., Huang J.C., Zhang W., Dong Z.H., Yang Y.P. (2024). Occurrence of *Panaphis juglandis*, the damage caused by this pest, and the potential for it to be controlled by its dominant naturalenemy, *Calvia muiri*. Chin. J. Appl. Entomol..

[36] Chen B., Chen W.B., Xue C.Z., Li Y.Y., Shen Z.J., Wang M.Q., Mao J.J., Dong H., Zhang L.S. (2025). Predation capacity of Ddevelopmental stage *Arma chinensis* to *Spodoptera frugiperda* eggs. Chin. J. Biol. Control.

[37] Meng J.Y., Li Z.M., Dong X.L., Wang H.C., Tan X.F., Guo X.G. (2022). Predation capacity of *Arma chinensis* nymphs on 3rd to 5th instar larvae of *Spodoptera frugiperda*. Jiangsu Agric. Sci..

[38] Zhou L.J., Yang D.H., Hu Q.L., Shi L.Z., Cao H.Q., Li G.T., Jiang X.C. (2022). Prey selectivity and predatory functional response of *Harmonia axyridis* to *Spodoptera frugiperda* and *Rhopalosiphum padi*. Plant Prot..

[39] Cai Z.P., Zhang X.R., Xiao Y.L., Zhang J.P., Ge F. (2024). Functional response and predation preference of multicolored Asian lady beetle *Harmonia axyridis* to two aphids in the micro-landscape of apple and Monnier’s snowparsley *Cnidium monnieri*. J. Plant Prot..

[40] Hashimoto N., Yano E., Watanabe T., Hemerik L., Kandori L. (2025). Voracity of *Nesidiocoris tenuis* (Hemiptera: Miridae) for *Bemisia tabaci* (Hemiptera: Aleyrodidae) and *Thrips palmi* (Thysanoptera: Thripidae) and its functional response to the density of *B. tabaci*. Appl. Entomol. Zool..

[41] Rashedi A., Rajabpour A., Sohani N.Z., Rasekh A. (2020). Prey stage preference and functional response of *Orius albidipennis* (Hetetroptera, Anthocoridae) to *Aphis fabae* (Homomoptera, Aphididae). Int. J. Trop. Insect Sci..

[42] Huang Q., Long L.P., Huang S.S., Wu B.Q., Li C., Ling Y. (2025). Functional and numerical responses of *Tytthus chinensis* (Hemiptera: Miridae) to *Sogatella furcifera* (Hemiptera: Delphacidae). Insects.

[43] Li W.J., Yuan H., Lu Y.H., Li Q., Wu K.M. (2015). Diet selection of *Adelphocoris suturalis* on *Phaseolus vulgaris* pods and *Helicoverpa armigera* eggs. Plant Prot..

[44] Liang H.J., Li Y., Sun C.Y., Feng L.K., Wang P.L., Lu Y.H. (2013). The predation of *Lygus pratensis* (L.) to *Aphis gossypii* Glover. J. Environ. Entomol..

[45] Hassanpour M., Bagheri M., Golizadeh A., Farrokhi A. (2016). Functional response of *Nesidiocoris tenuis* (Hemiptera: Miridae) to *Trialeurodes vaporariorum* (Hemiptera: Aleyrodidae): Effect of different host plants. Biocontrol Sci. Technol..

[46] Perdikis D., Fantinou A., Lykouressis D. (2011). Enhancing pest control in annual crops by conservation of predatory Heteroptera. Biol. Control.

[47] Lu Y.H., Wu K.M., Wyckhuys A.K., Guo Y.Y. (2010). Overwintering hosts of *Apolygus lucorum* (Hemiptera: Miridae) in northern China. Crop Prot..

[48] Pan H.S., Liu B., Lu Y.H., Wyckhuys A.K. (2015). Seasonal alterations in host range and fidelity in the polyphagous mirid bug, *Apolygus lucorum* (Heteroptera: Miridae). PLoS ONE.

[49] Ma X.L., Song H.W., Feng H.Q., Zhang Z., Lu S.H., Yuan G.J., Li Z.Q. (2016). Migration abilities and host transfer rules of *Apolygus lucorum* Meyer-Dür in spring and autumn in Henan jujube area. J. Henan Agric. Sci..

[50] Pan H.S., Xiu C.L., Williams L., Lu Y.H. (2021). Plant volatiles modulate seasonal dynamics between hosts of the polyphagous mirid bug *Apolygus lucorum*. J. Chem. Ecol..

